# The interaction between systemic inflammation and psychosocial stress in the association with cardiac troponin elevation: A new approach to risk assessment and disease prevention

**DOI:** 10.1016/j.ypmed.2016.09.018

**Published:** 2016-12

**Authors:** Antonio Ivan Lazzarino, Mark Hamer, David Gaze, Paul Collinson, Ann Rumley, Gordon Lowe, Andrew Steptoe

**Affiliations:** a London School of Hygiene & Tropical Medicine, Keppel Street, London WC1E 7HT, United Kingdom; b Department of Epidemiology and Public Health, University College London, 1-19 Torrington Place, London WC1E 6BT, United Kingdom; c Chemical Pathology, Clinical Blood Sciences, St George's Healthcare NHS Trust, Blackshaw Road, Tooting, London SW17 0QT, United Kingdom; d Institute of Cardiovascular and Medical Sciences, University of Glasgow, Glasgow G12 8TA, United Kingdom; e School of Sport, Exercise, and Health Sciences, National Centre for Sport & Exercise Medicine, Loughborough University, LE11 3TU, United Kingdom

**Keywords:** Allostasis, Atherosclerosis, Cardiovascular diseases, Fibrinogen, Neuroendocrinology, Psychological stress, Socioeconomic factors, Troponin T

## Abstract

We have previously shown that there is a complex and dynamic biological interaction between acute mental stress and acute release of inflammatory factors into the blood stream in relation to heart disease. We now hypothesize that the presence of chronic psychosocial stress may modify the weight of single test results for inflammation as a predictor of heart disease. Using a cross-sectional design, 500 participants free from heart disease drawn from the Whitehall II study in UK in 2006–2008 were tested for plasma fibrinogen as an inflammatory factor, financial strain as a marker of chronic psychosocial stress, coronary calcification measured using computed tomography, and for plasma high-sensitivity cardiac troponin T (HS-CTnT) as a marker of cardiac risk. Fibrinogen concentration levels above the average were associated with a 5-fold increase in the odds of HS-CTnT positivity only among individuals with financial strain (N = 208, OR = 4.73, 95%CI = 1.67 to 13.40, P = 0.003). Fibrinogen was in fact not associated with HS-CTnT positivity in people without financial strain despite the larger size of that subsample (n = 292, OR = 0.84, 95%CI = 0.42 to 1.67, P = 0.622). A test for interaction on the full sample (N = 500) showed a P value of 0.010 after adjusting for a range of demographics, health behaviours, traditional cardiovascular risk factors, psychosocial stressors, inflammatory cytokines, and coronary calcification. In conclusion, elevated fibrinogen seems to be cardio-toxic only when is combined with financial strain. Chronic psychosocial stress may modify the meaning that we should give to single test results for inflammation. Further research is needed to confirm our results.

## Highlights

• Chronic psychosocial stress modifies the association between inflammation and CVD.
• A single test result of elevated fibrinogen can be the result of a healthy acute response to stress.
• Therefore a finding of elevated fibrinogen cannot always be interpreted as a sign of poor health.
• Elevated fibrinogen is associated with CVD only in individuals with chronic stress.

## 1 Introduction

Inflammation is recognised as a fundamental determinant of atherosclerosis and cardiovascular disease (CVD), which are now indeed labelled as inflammatory conditions (Epstein and Ross, 1999, Libby and Theroux, 2005, Libby, 2013).

Chronic psychosocial stressors such as anxiety and depression are associated with inflammation (Dimsdale, 2008, Lazzarino et al., 2013a, Lazzarino et al., 2013b, Lazzarino et al., 2013c, Steptoe and Vögele, 1991) and with heart disease with an effect size that is comparable to that of the traditional risk factors such as high blood pressure, cholesterol, smoking, etc. (Brotman et al., 2007, Dimsdale, 2008, Steptoe and Kivimäki, 2012, Kivimäki et al., 2012).

Although the mechanisms involved in these complex pathways of causation have yet to be clarified genetically and phenotypically, there have been attempts to add markers of stress and inflammation when performing cardiovascular risk assessments (Macleod et al., 2007, Schnohr et al., 2015, Fiscella et al., 2009, Pearson et al., 2003, Ioannidis and Tzoulaki, 2012). As yet, the novel biomarkers failed to add substantial predictive accuracy to the equations based on the traditional risk factors for cardiovascular disease (Boon et al., 2014, Emerging Risk Factors Collaboration et al., 2012, Goff et al., 2014). It has been argued that the linear, monophasic approaches used to explain these mechanisms are not appropriate, and that “until a paradigm shift is adopted, cardiovascular biomarker research may remain fascinating but probably unhelpful to medical practice and public health” (Ioannidis and Tzoulaki, 2012).

In clinical and research settings, chronic systemic inflammation is flagged using single blood tests for inflammatory biomarkers such as fibrinogen, C-reactive protein, and others. We hypothesize that single tests for inflammation may have failed to add accuracy to predictive models because the results from those tests may vary due to an individual's exposure to psychosocial stress (either an unhealthy chronic exposure or a healthy acute exposure). In fact, in a laboratory-based experiment we found that the plasma concentration of inflammatory biomarkers can change very rapidly (< 1 min) after one brief episode of mild mental stress (Lazzarino et al., 2015). The kind of artificial stress that we have induced to our study participants was similar to the frequent, mild, stressful events of everyday life. It is therefore arguable that the concentration of inflammatory biomarkers in the blood has a physiological continuous oscillation during anyone's everyday life. Such instability affects the appropriateness of using single blood tests to flag chronic systemic inflammation. Furthermore, we found a counterintuitive association between baseline inflammation and the extent of inflammatory reaction to acute stress: people with low baseline inflammation had sharp and high inflammatory responses to acute stress, and this phenotype was associated with low cardiac risk. Conversely, people with higher baseline inflammation had blunted and lower inflammatory responses and presented higher cardiac risk (Lazzarino et al., 2015). Therefore, a finding of elevated inflammation might be an indicator of chronic elevation due to chronic psychosocial stress but also an indicator of a healthy acute response to a recent acute stressful event. In other words, a finding of elevated inflammation would be clinically unfavourable only if it is coupled with chronic psychosocial stress, and favourable if it is due to a healthy stress response in an individual with good psychosocial adaptation. These dynamics make single tests for inflammation further difficult to interpret when not impossible.

There are many inter-connected indicators of chronic psychosocial stress used in the medical literature (Steptoe, 2007). Factors such as financial strain, job strain, and social network, can be measured objectively and represent someone's objective exposure to stress. On the other hand, factors such as depression and anxiety, although have more clinical meaning, are subjective mental representations. Nevertheless, there is evidence that low socioeconomic status is associated with depression and anxiety (Lazzarino et al., 2013a, Lazzarino et al., 2013b) and thus the option of using financial strain as a marker of chronic psychosocial stress has the advantage of being both an objective measure and that of being highly correlated with more clinical conditions such as anxiety and depression. We have preferred financial strain over employment grade because the latter may not necessarily imply psychosocial stress.

Fibrinogen is a plasma protein produced by the liver and is a major coagulation factor. It is a positive acute-phase reactant protein (i.e. its concentration increases with inflammation), it is traditionally considered as a risk factor for cardiac disease because it promotes formation of thrombus, and it is considered as an inflammatory cytokine (Lowe et al., 2004, Stulnig, 2013).

Cardiac Troponin T is a marker of myocardial cell damage that is routinely measured in the peripheral blood plasma for the diagnosis of acute myocardial infarction in clinical settings. A high-sensitivity assay (HS-CTnT) has recently been developed and in healthy people it is associated with greater incidence of heart disease, cardiovascular mortality, and all-cause mortality, and is therefore considered the most proximal sentinel marker of heart disease (de Lemos et al., 2010, deFilippi et al., 2010).

For those reasons the aim of this study was to determine if financial strain (marker of chronic psychosocial stress) modifies the association between single test results for fibrinogen (marker of inflammation) and positivity for plasma HS-CTnT (marker of cardiac risk), so that high financial strain would increase the effect size of that association.

## 2 Methods

### 2.1 Study design

This cross-sectional study involved participants drawn from the Whitehall II epidemiological cohort (Marmot et al., 1991) between 2006 and 2008 in the United Kingdom (Hamer et al., 2010). The criteria for entry into the study included no history or objective signs of clinical or subclinical cardiovascular disease, no previous diagnosis or treatment of hypertension, inflammatory diseases, allergies, or kidney disease. Cardiovascular disease was defined as prior myocardial infarction, stable or unstable angina, revascularization procedure, heart failure, transient ischaemic attack, stroke, or electrocardiographic abnormalities (resting 12-lead electrocardiograms were taken). Volunteers were of white European origin, aged 53–76 years, and 56.5% were in full-time employment. Selection was stratified by grade of employment (current or most recent) to include both higher and lower socioeconomic status participants. From the initially invited participants (n = 1169), 27.6% were not eligible (mainly due to use of prescribed medications) and 25.9% declined to take part. There was no evidence of selection bias due to those exclusions with respect to the known characteristics of the sample (demographics, health behaviours, and traditional cardiovascular risk factors). Participants were prohibited from using any anti-histamine or anti-inflammatory medication 7 days prior to testing and were rescheduled if they reported colds or other infections on the day of testing. Participants gave full informed consent to participate in the study and ethical approval was obtained from the University College London Hospital committee on the Ethics of Human Research.

### 2.2 Data collection

We carried out clinical examinations in a light- and temperature-controlled laboratory. Participants were instructed to refrain from drinking caffeinated beverages or smoking for at least 2 h before the study and not to have performed vigorous physical activity or consumed alcohol since the previous evening. Venipuncture was performed using a butterfly needle. Blood pressure was taken 30 min after needle insertion (using an automated UA-779 digital monitor), as well as saliva and fasting blood samples.

Financial strain was assessed with an adaptation of the economic strain measure of Pearlin et al. (1981). This assesses difficulty paying one's bills, being able to replace items such as furniture or a car when needed, and being able to provide for one's family in terms of food, clothing, and medical care. Eight items were presented, with response options ranging from 1 = no difficulty to 3 = very great difficulty (Cronbach's alpha = 0.86). Therefore possible values range from 8 (lowest financial strain) to 24 (highest financial strain) (Steptoe et al., 2005). Other psychosocial stressors were assessed, including depression symptoms as measured using the Centre for Epidemiologic Studies Depression Scale (CES-D) (Eaton et al., 2004), and mental quality of life as measured using the 36-item Short Form Health Survey (SF-36) (Ware and Sherbourne, 1992). We derived two scores from the SF-36: mental health (items 9b–d and 9f–h) and mental component (items 5, 6, 9, and 10). We also used the PANAS scale to measure positive and negative affect (Watson et al., 1988).

We determined plasma fibrinogen concentration by an automated Clauss assay in a MDA-180 coagulometer (Organon Teknika, Cambridge, UK) using the manufacturer's reagents and the International fibrinogen standard (Gaffney and Wong, 1992).

We measured cardiac troponin T plasma concentration using a highly sensitive assay on an automated platform (Elecsys 2010 Troponin T hs STAT, Roche Diagnostics) (Collinson, 2011, Giannitsis et al., 2010).

We assessed coronary artery calcification (CAC Agatston score) in separate sessions using electron beam computed tomography (GE Imatron C-150, San Francisco, CA, USA) as previously described (Anand et al., 2007).

### 2.3 Data analysis

We recoded fibrinogen as a Z-score or as a binary variable. We set two different cut-off points for the binary categorisation so that we could evaluate whether the interaction between fibrinogen and financial strain was consistently present at different levels of fibrinogen elevation. Cut-off points were set at 300 mg/dL (median and mean value) or at 400 mg/dL (established clinical upper limit for normal values) (Schmaier, 2012).

Financial strain was considered as a binary variable (0 = absent; 1 = present) with a cut-off using the minimum score of eight (58.4% of the sample scored eight). An interaction parameter was calculated by multiplying fibrinogen (Z-score) and financial strain (binary) together. Agatston CAC score had a right-skewed distribution and it was transformed into an ordered categorical variable with four categories (cut-offs at 0, 100, and 400). HS-CTnT was cut into a binary variable (detectable vs undetectable) because only about 17% of the sample had positive values.

We used multiple logistic regression models to examine the combined association of plasma fibrinogen concentration and financial strain with HS-CTnT. The interaction between fibrinogen and financial strain was firstly assessed by means of stratified analyses, which have the disadvantage of reducing the analytic sample size and the advantage of giving a visual impression of the interaction. We then assessed the interaction in the full sample using the multiplicative parameter as a covariate. We adjusted for demographics such as age, gender, and employment grade, and for health behaviours such as physical activity, smoking and alcohol intake because they are related to CVD and may confound the association between fibrinogen and HS-CTnT. We also took into account clinical variables that are known to be linked with CVD such as Body Mass Index (BMI), blood pressure, glycated haemoglobin (HbA1c), triglycerides, Low-Density Lipoprotein cholesterol (LDL), and total/High-Density Lipoprotein (HDL) cholesterol ratio. Moreover, we adjusted for circulating markers of inflammation and endothelial dysfunction such as High-Sensitivity C-Reactive Protein (HS-CRP), salivary cortisol, High-Sensitivity Interleukin-6 (HS-IL-6), von Willebrand Factor (vWF), and Monocyte Chemoattractant Protein-1 (MCP-1) to account for vascular inflammation and endothelial dysfunction. We also adjusted for chronic psychosocial stressors including CES-D score, SF-36 scores, positive affect, negative affect, to assess if financial strain was independent from them. Finally, we adjusted for Agatston CAC score to examine whether the interaction between fibrinogen and financial strain was independent of underlying coronary atherosclerosis.

Financial strain was considered a priori as the best candidate for the role of effect modifier, for the reasons explained in the Introduction. However, we carried out additional analyses to also test the other psychosocial variables for interaction.

Missing values were minimal (< 1%), were only present in the covariates, and were imputed using multiple chained equations (Royston and White, 2011). Complete case analysis gave very similar results (data shown in the Appendix). We evaluated the extent of multicollinearity among covariates by computing one-to-one correlation coefficients and by examining variance inflation factors (VIF) (“Regression with Stata Web Book: Chapter 2 - Regression Diagnostics,”, n.d.). We performed several sensitivity analyses: CAC was log-transformed (zero values were recoded to values equal to the half of the smallest value in the dataset) as well as other right-skewed covariates; SF-36 mental health and SF-36 mental component showed left-skewed distributions and we recoded their values to their reciprocal before the log-transformation. We used both the Wald test and the Likelihood Ratio test to assess the interactions.

A total of 543 people participated in the study, but 42 (7.7%) had missing information for HS-CTnT or fibrinogen due to insufficient blood samples and were therefore excluded. One participant had missing information for financial strain. The final analytic sample comprised 500 disease-free participants aged 63 years on average (standard deviation [SD] = 5.7) of whom 55.2% were men. The excluded participants did not differ significantly from the main sample on any of the covariates.

## 3 Results

Financial strain was present in 41.6% of our sample. The average concentration of fibrinogen in the peripheral blood plasma was 314 mg/dL (SD = 60.7), and 17.0% showed positivity for HS-CTnT in the peripheral blood plasma.

Table 1 describes the sample according to financial strain. The description of skewed variables is presented using medians and inter-quartile ranges (IQR). People with financial strain tended to have higher concentrations of fibrinogen, to have lower grades of employment, to be less physically active, to have lower HDL cholesterol, and to score poorly in other psychosocial measures.

Table 2 shows the output from several logistic regression models run after stratification for financial strain. After adjusting for demographic variables, fibrinogen concentration levels above the average were associated with a 5-fold increase in the odds of HS-CTnT positivity only among individuals with financial strain (N = 208, OR = 4.73, 95%CI = 1.67 to 13.40, P = 0.003). Fibrinogen was not associated with HS-CTnT positivity in people without financial strain, despite the larger size of that subsample (n = 292, OR = 0.84, 95%CI = 0.42 to 1.67, P = 0.622). Further multiple adjustments did not change the pattern of results noticeably. The results were also similar when we applied an alternative cut-off point to mark elevated fibrinogen (400 mg/dL instead of the average 300 mg/dL).

Table 3 shows unstratified analyses, in which the synergy between financial strain and fibrinogen was tested using an interaction parameter as a covariate in the full sample. There was very strong evidence of an interaction after adjusting for age and gender (OR = 2.09, 95%CI = 1.25 to 3.52, P = 0.005). The strength of association did not decrease after further multiple adjustments, although the extent of statistical evidence decreased but remained significant (OR = 2.07, 95%CI = 1.19 to 3.60, P = 0.010).

Table 4 shows the full output from the fully-adjusted multiple logistic regression model. Fibrinogen and financial strain did not show any association with HS-CTnT, but there was a clear interaction between these variables (P = 0.010), therefore fibrinogen may be a risk factor for HS-CTnT elevation only when financial strain is present. No other covariates in the model showed any evidence of association with the outcome apart from age (P < 0.001) and gender (P < 0.001).

None of other psychosocial variables (CES-D, SF-36 mental health, SF-36 mental component, positive affect, and negative affect) showed any evidence of interaction with fibrinogen when they replaced financial strain.

The exclusion of one or more of those covariates showing multicollinearity (correlation coefficient > 0.35 or VIF < 3) in rotation and/or in combination did not alter the results distinctively. All sensitivity analyses gave similar results to the main analyses.

## 4 Discussion

Our results indicate that a single test result of elevated fibrinogen might be associated with higher risk of cardiac disease only when it is accompanied with financial strain.

We are therefore proposing a new approach to cardiovascular risk assessment: inflammation and psychosocial stress may interact with each other in the promotion of heart disease. Our results may help understand why inflammatory biomarkers are not able to improve the prediction of cardiac events in spite of inflammation being the necessary precursor of heart disease: since the concentration of inflammatory biomarkers in the peripheral blood plasma changes very rapidly in response to changes in environmental factors such as acute stress, the presence or absence of chronic stress modifies the weight that we should give to normal or abnormal test results for inflammation. Our results suggest that in some instances such weight may even be overturned, i.e. that abnormal test results may be associated with favourable outcomes. Therefore an accurate risk assessment based on single tests for inflammatory biomarkers becomes possible only after taking environmental factors into account in an appropriate way.

We have used one established marker for psychosocial stress and one for systemic inflammation, but both stress and inflammation are umbrella terms for a range of indicators and interconnected markers within each domain, and each indicator measures different but often related aspects of each phenomenon, thus future research must focus on the identification and on the isolation of the most important markers, i.e. those that show the highest degree of interaction. We found an interaction between stress and inflammation only when we used financial strain as a marker of stress. However, we cannot be sure that financial strain is intrinsically more valuable or more able to capture certain important psychosocial aspects compared to the other available markers; financial strain has the advantage of being an objective measure and therefore it may just be a more accurate and more efficient marker compared to the others from a statistical perspective. Interaction tests are in fact known to have low power and therefore require large samples and very accurate measures. Hence, we cannot rule out the hypothesis that if these analyses were replicated on a larger sample, the other stress markers would similarly show interaction with inflammation. Future research may also examine other markers of inflammation.

Our outcome was HS-CTnT, which is a recognised indicator of myocardial damage and the most proximal sentinel marker for cardiac disease, but it is necessary to challenge our results using a recorded clinical event as an outcome.

Regarding sample size, interaction tests are known to require large sample sizes to have sufficient statistical power and this fact further strengthens our results achieved with a relatively small sample. However, the precision of the estimates might be improved if these analyses were applied on a larger sample, as some of our estimates showed large confidence intervals.

No other covariates in our full model showed good evidence of association with the outcome (apart from age and gender, which are known determinants of heart disease), and the variable measuring the stress-inflammation interaction was by far the strongest in terms of effect size and statistical significance. Therefore the effect of stress-inflammation interactions may be higher than those of established risk factors for heart disease such as blood pressure and cholesterol, and moreover the interaction appeared to be very robust and independent from other factors since its coefficient did not change after a number of multiple stepwise adjustments (Table 3).

The prevalence of detectable HS-CTnT in our sample (17.0%) was similar to that reported in a nationally-representative CVD-free population sample in USA (15.7%) (de Lemos et al., 2010), and our normal range (mean ± 1.96*SD) for plasma fibrinogen was 195 to 433 mg/dL, which almost perfectly overlaps with the reference range for normal values used in clinical practice (200 to 400 mg/dL) (Schmaier, 2012). These findings suggest that the selection of participants was unbiased.

Our study involved participants free from cardiovascular disease because we are interested in the primary prevention of heart disease. This approach has the disadvantage of generating results that are not necessarily applicable to the general population or to high-risk groups. Therefore analyses on wider and more representative population groups are necessary to generalise our results.

This is a cross-sectional study and therefore we cannot determine the causal sequence. Stress-inflammation interactions may contribute to early signs of heart disease, or people at an early stage of cardiac disease may be more prone to disturbed stress-inflammation interactions. If these findings were confirmed in larger, prospective studies against robust cardiovascular events, they might open new approaches to cardiovascular risk assessment in medical research. The results from a single blood measure are not ideal and a continuous monitoring of these parameters in conjunction with environmental factors seem more appropriate, something that might become possible with the advent of new technological devices able to capture real-time data (either self-reported or physiological) in synchrony with biological markers.

The results from our research suggest that a finding of elevated plasma fibrinogen may be the result of a healthy acute response to stress and therefore it cannot always be interpreted as a marker of poorer health. Moreover, there may be other reasons for a finding of elevated inflammation, which also may be or may be not associated with chronic inflammation and disease, including genetic, metabolic, and personality traits. Future research should take those possibilities into consideration.

No participants in our study reported acute mental stress reaction to the needle insertion and the blood draw. We think that this due to the fact that all our participants were volunteers and were informed about the procedure prior to the testing sessions. Therefore it is highly likely that none of them was prone to overreacting to the acute stress caused by a needle insertion and a blood draw. To the contrary, during real-life clinical assessments some people may overreact to that kind of acute stress and may release inflammatory factors as well as cardiac troponin in response to the perceived stress. This phenomenon can lead to wrong diagnosis.

In conclusion, elevated fibrinogen seems to be cardio-toxic only when is combined with financial strain. The presence of chronic psychosocial stress may modify the weight that we should give to single test results for inflammation. Further research is needed to confirm our results.

## Transparency document

Transparency document. Image 1

## Acknowledgments and disclosures

This research was supported by the British Heart Foundation (grant code RG/10/005/28296), United Kingdom. The funders played no role in any phase of the study. All authors declare no conflict of interest of any kind. All authors have made substantial contribution to the conception and design of the study, the acquisition of data, the interpretation of the results, and the critical review of the article. AIL drafted the article and carried out the data analysis and takes responsibility for the accuracy of the analysis. All authors have approved the final article.

## Figures and Tables

**Table 1:** Characteristics of the study sample by categories of financial strain for 500 disease-free participants drawn from the Whitehall II epidemiological cohort between 2006 and 2008 in United Kingdom.

Factor and category	Financial strain				P
	No		Yes		
	(n = 292)		(n = 208)		
Fibrinogen (mean mg/dL ± SD)	307.4	± 56.5	322.1	± 65.3	0.008
Age (mean years ± SD)	62.9	± 5.4	63.1	± 6.1	0.741
Male (%)	55.1		55.3		0.973
Latest grade of employment (%)					< 0.001
Higher	46.2		28.4		
Intermediate	36.3		44.2		
Lower	17.5		27.4		
Current smoker (%)	4.5		6.3		0.374
Alcohol consumption (%)					0.633
No alcohol	13.0		19.2		
Below recommended levels	75.0		64.9		
Above recommended levels	12.0		15.9		
Hours of physical activity per week (%)					0.073
< 1 h	21.7		25.3		
1–4 h	30.4		35.2		
5–7 h	23.8		21.3		
> 7 h	24.1		18.3		
Body mass index (mean Kg/m^2^ ± SD)	25.6	± 3.8	26.1	± 3.9	0.179
Systolic blood pressure (mean mm Hg ± SD)	128.3	± 15.7	130.1	± 15.8	0.221
Diastolic blood pressure (mean mm Hg ± SD)	69.6	± 8.9	69.9	± 8.5	0.694
Glycated haemoglobin (mean % ± SD)	5.5	± 0.5	5.5	± 0.4	0.659
Triglycerides (median g/L ± IQR)	1.1	± 0.8	1.2	± 0.7	0.974
HDL (mean mmol/L ± SD)	1.7	± 0.5	1.6	± 0.4	0.047
LDL (mean mmol/L ± SD)	3.0	± 0.9	3.0	± 0.8	0.591
Total cholesterol (mean mmol/L ± SD)	5.3	± 0.9	5.3	± 0.9	0.692
Total cholesterol/HDL ratio (mean ± SD)	3.3	± 1.1	3.4	± 1.0	0.284
HS-CRP (median mg/L ± IQR)	1.0	± 1.3	1.0	± 1.6	0.149
Salivary cortisol (mean nmol/L ± SD)	6.5	± 4.1	6.6	± 4.8	0.681
HS-IL-6 (median pg/ml ± IQR)	1.1	± 0.8	1.2	± 0.9	0.537
Von Willebrand factor (mean % ± SD)	104.2	± 39.6	105.8	± 39.9	0.648
MCP-1 (median pg/mL ± IQR)	137.2	± 60.9	138.4	± 53.5	0.431
CES-D (median score ± IQR)	4.0	± 7.0	6.0	± 8.0	0.002
SF-36 mental health (median score ± IQR)	84.0	± 16.0	80.0	± 20.0	0.001
SF-36 mental component (median score ± IQR)	85.1	± 10.8	82.0	± 16.6	< 0.001
Positive affect (median score ± IQR)	25.0	± 8.0	22.0	± 8.0	< 0.001
Negative affect (median score ± IQR)	2.0	± 4.0	4.0	± 5.0	0.001
Agatston coronary calcium score (%)					0.954
None	42.5		44.7		
< 100	32.9		31.7		
< 400	16.4		12.5		
400 +	8.2		11.1		
HS-CTnT detectable (%)	17.5		16.3		0.743
HS-CTnT concentration if detectable (median ng/L ± IQR)	5.6	± 3.9	5.8	± 4.4	0.661

SD = standard deviation. IQR = interquartile range. P values were computed using the likelihood ratio test. Ordered categorical variables such as employment grade, alcohol consumption, physical activity, and coronary calcium score were treated as linear (test for trend).

**Table 2:** Multiple logistic regression models for the association between plasma fibrinogen concentrations and plasma detectable HS-CTnT by strata of financial strain.

Subgroup	N	Binary exposure variable	Type of multiple logistic regression model for HS-CTnT positivity								
			Adjusted for demographics^a^			Further adjusted for health behaviours, cardiovascular risk factors, and psychosocial stressors^b^			Full model (further adjusted for inflammatory cytokines and coronary calcification)^c^		
			OR	(95% CI)	P	OR	(95% CI)	P	OR	(95% CI)	P
Low financial strain	292	Fibrinogen (> 300 vs ≤ 300 mg/dL)	0.84	(0.42 to 1.67)	0.622	0.87	(0.40 to 1.90)	0.735	0.91	(0.40 to 2.08)	0.820
High financial strain	208	Fibrinogen (> 300 vs ≤ 300 mg/dL)	4.73	(1.67 to 13.40)	0.003	4.82	(1.43 to 16.22)	0.011	3.86	(1.02 to 14.53)	0.046
Low financial strain	292	Fibrinogen (> 400 vs ≤ 400 mg/dL)	0.40	(0.08 to 2.00)	0.263	0.29	(0.05 to 1.67)	0.167	0.26	(0.04 to 1.91)	0.186
High financial strain	208	Fibrinogen (> 400 vs ≤ 400 mg/dL)	3.90	(1.22 to 12.49)	0.022	4.43	(1.05 to 18.68)	0.043	2.69	(0.55 to 13.12)	0.221

a Adjusted for age, gender, and latest grade of employment.

b Further adjusted for CES-D, SF-36 mental health, SF-36 mental component, positive affect, negative affect, smoking, alcohol consumption, physical activity, systolic and diastolic blood pressure, LDL, total cholesterol/HDL ratio, triglycerides, glycated haemoglobin, and BMI.

c Further adjusted for HS-CRP, salivary cortisol, HS-IL-6, vWF, MCP-1, and Agatston coronary calcification score.

**Table 3:** Multiple logistic regression models for the association of plasma fibrinogen concentration, financial strain, and an interaction parameter with plasma detectable HS-CTnT (n = 500).

Model for detectable HS-CTnT	Mutually-adjusted exposure variables	OR	(95%CI)	P
1. No further adjustments	Fibrinogen	0.98	(0.70 to 1.36)	0.893
	Financial strain	0.80	(0.48 to 1.34)	0.401
	Interaction	1.63	(1.02 to 2.61)	0.043
2. Further adjusted for age and gender	Fibrinogen	0.85	(0.59 to 1.21)	0.363
	Financial strain	0.77	(0.44 to 1.36)	0.373
	Interaction	2.09	(1.25 to 3.52)	0.005
3. Further adjusted for latest grade of employment	Fibrinogen	0.83	(0.58 to 1.19)	0.318
	Financial strain	0.75	(0.42 to 1.32)	0.312
	Interaction	2.07	(1.23 to 3.47)	0.006
4. Further adjusted for CES-D, SF-36 mental health, SF-36 mental component, positive affect, and negative affect	Fibrinogen	0.84	(0.58 to 1.21)	0.354
	Financial strain	0.77	(0.43 to 1.40)	0.397
	Interaction	2.07	(1.23 to 3.51)	0.007
5. Further adjusted for smoking, alcohol consumption, and physical activity	Fibrinogen	0.84	(0.58 to 1.22)	0.366
	Financial strain	0.77	(0.43 to 1.39)	0.390
	Interaction	2.07	(1.27 to 3.52)	0.007
6. Further adjusted for systolic and diastolic blood pressure, LDL, total cholesterol/HDL ratio, triglycerides, glycated haemoglobin, and BMI	Fibrinogen	0.80	(0.55 to 1.16)	0.236
	Financial strain	0.78	(0.43 to 1.44)	0.432
	Interaction	2.07	(1.20 to 3.55)	0.009
7. Further adjusted for HS-CRP, salivary cortisol, HS-IL-6, vWF, and MCP-1	Fibrinogen	0.72	(0.47 to 1.09)	0.117
	Financial strain	0.80	(0.43 to 1.47)	0.475
	Interaction	2.11	(1.22 to 3.67)	0.008
8. Further adjusted for Agatston coronary calcification score with 4 categories	Fibrinogen	0.71	(0.47 to 1.07)	0.105
	Financial strain	0.80	(0.43 to 1.48)	0.473
	Interaction	2.07	(1.19 to 3.60)	0.010

The OR pertaining to fibrinogen is for one standard deviation increase from the mean (Z score). Financial strain is a binary variable (1 vs 0). Interaction = multiplication between fibrinogen and financial strain.

**Table 4:** Full output from the final fully-adjusted multiple logistic regression model for the association of plasma fibrinogen concentrations, financial strain, and their interaction with plasma detectable HS-CTnT (n = 500).

Exposure variables	Mutually-adjusted OR for detectable HS-CTnT	(95%CI)	P
Fibrinogen	0.71	(0.47 to 1.07)	0.105
Financial strain	0.80	(0.43 to 1.48)	0.473
Interaction fibrinogen X financial strain	2.07	(1.19 to 3.60)	0.010
Age	1.19	(1.12 to 1.26)	< 0.001
Male	5.34	(2.51 to 11.36)	< 0.001
Latest grade of employment	1.16	(0.77 to 1.73)	0.478
CES-D	0.98	(0.91 to 1.07)	0.706
SF-36 mental health	1.01	(0.97 to 1.05)	0.769
SF-36 mental component	0.98	(0.94 to 1.02)	0.263
Positive affect	0.99	(0.94 to 1.04)	0.580
Negative affect	0.91	(0.82 to 1.02)	0.105
Alcohol consumption	0.90	(0.52 to 1.54)	0.693
Physical activity	0.93	(0.71 to 1.22)	0.613
Smoking	1.09	(0.24 to 5.00)	0.911
Systolic blood pressure	1.00	(0.97 to 1.02)	0.841
Diastolic blood pressure	1.00	(0.95 to 1.05)	0.944
LDL	1.13	(0.68 to 1.88)	0.624
Total cholesterol/HDL ratio	0.91	(0.53 to 1.55)	0.721
Glycated haemoglobin	1.41	(0.81 to 2.45)	0.221
BMI	1.05	(0.96 to 1.15)	0.288
Triglycerides	0.55	(0.28 to 1.06)	0.072
HS-IL-6	0.99	(0.66 to 1.48)	0.970
HS-CRP	1.06	(0.92 to 1.23)	0.416
Cortisol	1.03	(0.96 to 1.09)	0.416
vWF	1.00	(1.00 to 1.01)	0.203
MCP-1	1.00	(0.99 to 1.00)	0.602
Agatston coronary calcification score	1.28	(0.96 to 1.71)	0.091

The OR pertaining to fibrinogen is for one standard deviation increase from the mean (Z score). Financial strain is a binary variable (1 vs 0). Interaction = multiplication between fibrinogen and financial strain. The ORs pertaining to the other variables are for one unit increase, except for ordered categorical variables employment grade, alcohol consumption, physical activity, and coronary calcification score, for which the ORs are for one category increase.

## Appendix A Supplementary data

Supplementary tables Image 1
