# Supplementary material for: The interaction between systemic inflammation and psychosocial stress in the association with cardiac troponin elevation: A new approach to risk assessment and disease prevention
Source: Prev Med. 2016 Dec;93:46–52. doi: 10.1016/j.ypmed.2016.09.018 (PMC5126095; doi:10.1016/j.ypmed.2016.09.018)
Supplement: Supplementary file 1 — Transparency document. [file mmc1.docx]

Table 5. Multiple logistic regression models for the association between plasma fibrinogen concentrations and plasma detectable HS-CTnT by strata of financial strain, carried out without imputation of missing values. This table parallels Table 2 (imputed missing values).

| **Subgroup** | **Binary exposure variable** | **Type of multiple logistic regression model for detectable HS-CTnT** | | | | | | | | | | | | | | | | | |
| --- | --- | --- | --- | --- | --- | --- | --- | --- | --- | --- | --- | --- | --- | --- | --- | --- | --- | --- | --- |
|  |  |  |  |  |  |  |  |  |  |  |  |  |  |  |  |  |  |  |  |
|  |  | Adjusted for demographics* | | | | | | Further adjusted for health behaviours, cardiovascular risk factors, and psychosocial stressors† | | | | | | Full model (further adjusted for inflammatory cytokines and coronary calcification)‡ | | | | | |
|  |  |  |  |  |  |  |  |  |  |  |  |  |  |  |  |  |  |  |  |
|  |  |  |  |  |  |  |  |  |  |  |  |  |  |  |  |  |  |  |  |
|  |  |  |  |  |  |  |  |  |  |  |  |  |  |  |  |  |  |  |  |
|  |  | N | OR | (95% CI) | | | P | N | OR | (95% CI) | | | P | N | OR | (95% CI) | | | P |
| Low financial strain | Fibrinogen (>300 vs ≤300 mg/dL) | 292 | 0.84 | (0.42 | to | 1.67) | 0.622 | 268 | 0.86 | (0.39 | to | 1.90) | 0.707 | 247 | 0.60 | (0.23 | to | 1.56) | 0.296 |
| High financial strain | Fibrinogen (>300 vs ≤300 mg/dL) | 208 | 4.73 | (1.67 | to | 13.40) | 0.003 | 189 | 4.49 | (1.28 | to | 15.72) | 0.019 | 172 | 3.63 | (0.84 | to | 15.61) | 0.083 |
| Low financial strain | Fibrinogen (>400 vs ≤400 mg/dL) | 292 | 0.40 | (0.08 | to | 2.00) | 0.263 | 268 | 0.14 | (0.01 | to | 1.41) | 0.096 | 247 | 0.12 | (0.01 | to | 2.29) | 0.159 |
| High financial strain | Fibrinogen (>400 vs ≤400 mg/dL) | 208 | 3.90 | (1.22 | to | 12.49) | 0.022 | 189 | 5.21 | (1.17 | to | 23.14) | 0.030 | 172 | 2.14 | (0.31 | to | 14.71) | 0.441 |

*Adjusted for age, gender, and latest grade of employment. †Further adjusted for CESD score, SF36 mental health score, SF36 summary mental health status, positive affect, negative affect, smoking, alcohol consumption, physical activity, systolic and diastolic blood pressure, LDL, total cholesterol/HDL ratio, triglycerides, glycated haemoglobin, and BMI. ‡Further adjusted for HS-CRP, salivary cortisol, HS-IL6, vWF, MCP-1, and Agatston coronary calcification score.

Table 6. Multiple logistic regression models for the association of plasma fibrinogen concentration, financial strain, and an interaction parameter with plasma detectable HS-CTnT, carried out without imputation of missing values. This table parallels Table 3 (imputed missing values).

| **Model for detectable HS-CTnT** | **Mutually-adjusted** | **OR** | **(95%CI)** | | | **P** |
| --- | --- | --- | --- | --- | --- | --- |
|  | **exposure variables** |  |  |  |  |  |
| 1. No further adjustments (n=500) | Fibrinogen | 0.98 | (0.70 | to | 1.36) | 0.893 |
|  | Financial strain | 0.80 | (0.48 | to | 1.34) | 0.401 |
|  | Interaction | 1.63 | (1.02 | to | 2.61) | 0.043 |
| 2. Further adjusted for age and gender (n=500) | Fibrinogen | 0.85 | (0.59 | to | 1.21) | 0.363 |
|  | Financial strain | 0.77 | (0.44 | to | 1.36) | 0.373 |
|  | Interaction | 2.09 | (1.25 | to | 3.52) | 0.005 |
| 3. Further adjusted for latest grade of employment (n=500) | Fibrinogen | 0.83 | (0.58 | to | 1.19) | 0.318 |
|  | Financial strain | 0.75 | (0.42 | to | 1.32) | 0.312 |
|  | Interaction | 2.07 | (1.23 | to | 3.47) | 0.006 |
| 4. Further adjusted for CESD score, SF36 mental health score, SF36 summary mental health status, positive affect, and negative affect (n=494) | Fibrinogen | 0.84 | (0.58 | to | 1.21) | 0.350 |
|  | Financial strain | 0.78 | (0.43 | to | 1.41) | 0.415 |
|  | Interaction | 2.11 | (1.24 | to | 3.58) | 0.006 |
| 5. Further adjusted for smoking, alcohol consumption, and physical activity (n=482) | Fibrinogen | 0.81 | (0.56 | to | 1.18) | 0.281 |
|  | Financial strain | 0.82 | (0.45 | to | 1.49) | 0.521 |
|  | Interaction | 2.18 | (1.27 | to | 3.76) | 0.005 |
| 6. Further adjusted for systolic and diastolic blood pressure, LDL, total cholesterol/HDL ratio, triglycerides, glycated haemoglobin, and BMI (n=457) | Fibrinogen | 0.78 | (0.53 | to | 1.15) | 0.212 |
|  | Financial strain | 0.79 | (0.42 | to | 1.48) | 0.463 |
|  | Interaction | 2.19 | (1.23 | to | 3.89) | 0.007 |
| 7. Further adjusted for HS-CRP, salivary cortisol, HS-IL6, vWF, and MCP-1 (n=419) | Fibrinogen | 0.68 | (0.42 | to | 1.09) | 0.111 |
|  | Financial strain | 0.90 | (0.46 | to | 1.75) | 0.748 |
|  | Interaction | 2.26 | (1.19 | to | 4.32) | 0.013 |
| 8. Further adjusted for Agatston coronary calcification score with 4 categories (n=419) | Fibrinogen | 0.67 | (0.42 | to | 1.08) | 0.102 |
|  | Financial strain | 0.89 | (0.45 | to | 1.74) | 0.728 |
|  | Interaction | 2.21 | (1.14 | to | 4.25) | 0.018 |

The OR pertaining to fibrinogen is for one standard deviation increase from the mean (Z score). Financial strain is a binary variable (1 vs 0). Interaction = multiplication between fibrinogen and financial strain.

Table 6. Full output from the final fully-adjusted multiple logistic regression model for the association of plasma fibrinogen concentrations, financial strain, and their interaction with plasma detectable HS-CTnT, carried out without imputation of missing values. This table parallels Table 4 (imputed missing values).

| **Exposure variables** | **Mutually-adjusted OR for detectable HS-CTnT** | **(95%CI)** | | | **P** |
| --- | --- | --- | --- | --- | --- |
|  |  |  |  |  |  |
|  |  |  |  |  |  |
| Fibrinogen | 0.67 | (0.42 | to | 1.08) | 0.102 |
| Financial strain | 0.89 | (0.45 | to | 1.74) | 0.728 |
| Interaction fibrinogen X financial strain | 2.21 | (1.14 | to | 4.25) | 0.018 |
| Age | 1.21 | (1.13 | to | 1.28) | <0.001 |
| Male | 6.09 | (2.51 | to | 14.77) | <0.001 |
| Latest grade of employment | 1.23 | (0.79 | to | 1.93) | 0.356 |
| CESD score | 1.00 | (0.91 | to | 1.10) | 0.986 |
| SF36 mental health score | 1.00 | (0.96 | to | 1.05) | 0.824 |
| SF36 summary mental health status | 0.99 | (0.95 | to | 1.03) | 0.604 |
| Positive affect | 0.98 | (0.93 | to | 1.04) | 0.515 |
| Negative affect | 0.89 | (0.79 | to | 1.00) | 0.059 |
| Alcohol consumption | 0.92 | (0.50 | to | 1.68) | 0.784 |
| Physical activity | 0.92 | (0.68 | to | 1.24) | 0.576 |
| Smoking | 1.52 | (0.32 | to | 7.32) | 0.601 |
| Systolic blood pressure | 0.99 | (0.96 | to | 1.02) | 0.657 |
| Diastolic blood pressure | 1.01 | (0.95 | to | 1.06) | 0.788 |
| LDL | 1.19 | (0.67 | to | 2.12) | 0.554 |
| Total cholesterol/HDL ratio | 0.74 | (0.40 | to | 1.36) | 0.328 |
| Glycated haemoglobin | 1.10 | (0.44 | to | 2.74) | 0.839 |
| BMI | 1.09 | (0.98 | to | 1.21) | 0.098 |
| Triglycerides | 0.74 | (0.36 | to | 1.49) | 0.393 |
| HS-IL6 | 0.87 | (0.55 | to | 1.36) | 0.538 |
| HS-CRP | 1.07 | (0.91 | to | 1.26) | 0.388 |
| Cortisol | 1.04 | (0.97 | to | 1.12) | 0.223 |
| vWF | 1.00 | (1.00 | to | 1.01) | 0.360 |
| MCP-1 | 1.00 | (0.99 | to | 1.00) | 0.757 |
| Agatston coronary calcification score | 1.33 | (0.97 | to | 1.83) | 0.078 |

The OR pertaining to fibrinogen is for one standard deviation increase from the mean (Z score). Financial strain is a binary variable (1 vs 0). Interaction = multiplication between fibrinogen and financial strain. The ORs pertaining to the other variables are for one unit increase, except for ordered categorical variables employment grade, alcohol consumption, physical activity, and coronary calcification score, for which the ORs are for one category increase.
