# Supplementary material for: The interaction between systemic inflammation and psychosocial stress in the association with cardiac troponin elevation: A new approach to risk assessment and disease prevention
Source: Prev Med. 2016 Dec;93:46–52. doi: 10.1016/j.ypmed.2016.09.018 (PMC5126095; doi:10.1016/j.ypmed.2016.09.018)
Supplement: Supplementary file 2 — Supplementary tables [file mmc2.pdf]

# Corresponding Author's Declaration Form
## *Preventive Medicine*

| | |
|---|---|
| Manuscript title: | The interaction between systemic inflammation and psychosocial stress in the association with cardiac troponin elevation: a new approach to risk assessment. |
| Corresponding author: | Antonio Ivan Lazzarino |
| Additional authors in the order provided in the manuscript: | Mark Hamer, David Gaze, Paul Collinson, Ann Rumley, Gordon Lowe, Andrew Stepto. |

**The corresponding author must provide statements of authorship, originality, conflicts of interest, and research funding on behalf of all authors of the manuscript.**

**Authorship and Originality:**
The corresponding author warrants that all aforementioned authors fulfill the criteria of authorship as defined by the International Committee of Medical Journal Editors (ICMJE) and explained here. The corresponding author further warrants that the work described in this manuscript has not been published before and is not (nor will be) under consideration elsewhere while under review in *Preventive Medicine*; that all authors approved the present submitted version and their institutions have no objections to the manuscript's contents.

**Conflict of interest:**
The corresponding author submits the following disclosure of financial or other relationships with companies or organizations that are stakeholders in the topic of the manuscript. For details read this fact sheet. Choose one below:

| | |
|---|---|
| ✔ | The authors have no conflicts of interest to disclose |
| ☐ | The following authors report specific relationships (name author, nature of the relationship, and company or organization): |

**Funding source:**
All sources of funding that could have influenced or could be perceived to influence the outcome of this work should be acknowledged. You as corresponding author should declare any involvement of study sponsors in the study design; collection, analysis and interpretation of data; the writing of the manuscript; the decision to submit the manuscript for publication. If the study sponsors had no such involvement, this should be stated. Choose one below:

| | |
|---|---|
| ✔ | There were no sources of funding that could have influenced the outcome of this work |
| ☐ | The following authors report financial support (name author, granting organization, grant number, or company sponsoring the study): |

**The corresponding author signed this statement on behalf of all coauthors to indicate that the above information is true, correct and complete.**

| | |
|---|---|
| Signature: Antonio Ivan Lazzarino (Digitally signed by Antonio Ivan Lazzarino DN: cn=Antonio Ivan Lazzarino, o, ou, email=a.i.lazzarino@gmail.com, c=GB Date: 2016.05.23 13:52:59 +01'00') | Print name: Antonio Ivan Lazzarino |
| | Date: 23rd May 2016 |

(This form is fillable with adobe software or equivalent. Both graphic and electronic validation signatures via software are acceptable. Alternatively you can sign by hand. Please upload the signed copy with your submission.)
